# BioMThermDB 1.0: Thermophysical Database of Proteins in Solutions

**DOI:** 10.3390/ijms232315371

**Published:** 2022-12-06

**Authors:** Mina Nikolić, Sandi Brudar, Evangelos Coutsias, Ken A. Dill, Miha Lukšič, Carlos Simmerling, Barbara Hribar-Lee

**Affiliations:** 1 Faculty of Chemistry and Chemical Technology, University of Ljubljana, Večna Pot 113, SI-1000 Ljubljana, Slovenia; 2 Laufer Center for Physical and Quantitative Biology, Stony Brook University, Stony Brook, NY 11794-5252, USA

**Keywords:** database, proteins, solutions, thermodynamics, hydrodynamic radius, electrophoretic mobility, zeta potential, diffusion coefficient, viscosity, cloud-point temperature

## Abstract

We present here a freely available web-based database, called BioMThermDB 1.0, of thermophysical and dynamic properties of various proteins and their aqueous solutions. It contains the hydrodynamic radius, electrophoretic mobility, zeta potential, self-diffusion coefficient, solution viscosity, and cloud-point temperature, as well as the conditions for those determinations and details of the experimental method. It can facilitate the meta-analysis and visualization of data, can enable comparisons, and may be useful for comparing theoretical model predictions with experiments.

## 1. Introduction

Proteins are the most abundant macromolecules in living cells and represent the building blocks of life. They govern almost all biological processes that define living organisms the way they are. Knowledge of the physical properties of protein solutions can have practical importance for formulating biological agents and drugs [1,2]. In order to maintain their beneficial functions, proteins must remain stable in environments in which they are immersed, usually in different aqueous solutions. Consequently, a wide and diverse set of information on the thermophysical and thermodynamic properties of proteins in aqueous solutions is of critical importance for obtaining a better understanding of the protein structure and its relationship with factors that influence its stability, which is vital for preparing safe pharmaceutical formulations. With the development of biophysical methods for protein characterization and web-based applications, bio-macromolecular studies have become extremely data-rich; thus, the need for data storage, its organization, and interconnection is increasing rapidly these days. Even though a great amount of useful information is available in existing databases, such as ProThermDB [3], MPTherm [4], PROXiMATE [5], and PINT [6], there remains an unmet need for specific data to answer everyday questions that arise in the preparation and modeling of protein solutions; e.g., there are questions such as what will be the phase stability and approximate viscosity of a given protein solution, what type of interactions can be expected in a particular protein solution, and how do its properties change with modifying conditions, such as protein concentration, pH, and temperature. In this study, we developed a database for protein and antibody solutions, called BioMThermDB 1.0, which consists of a broad set of thermophysical and dynamic properties that can help provide adequate answers for such puzzles. The obtained database was created by gathering data from both comprehensive and often scattered scientific literature, as well as from our own experimental results. The database is web-based and enables its users to obtain frequently elusive numerical values of thermophysical quantities. The database is freely available at https://phys-biol-modeling.fkkt.uni-lj.si/biomthermdb.html (current version of 5 October 2022).

## 2. Results and Discussion

**Figure 1:**
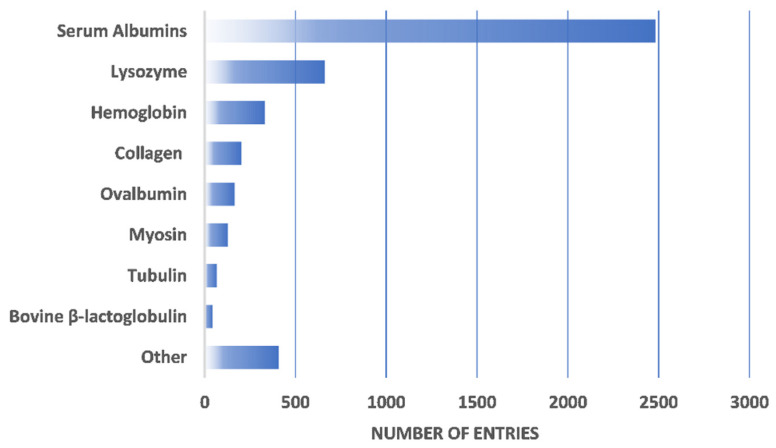
Distribution of thermophysical data entries based on their protein family.

BioMThermDB 1.0 provides thermophysical and thermodynamic data predominantly for globular proteins (e.g., various serum albumins, lysozyme, hemoglobin, etc.) and antibodies but information about other proteins is also available (see Figure 1 and Table 1). Each entry is given as a specific protein solution that contains information about the overall composition of the solution; this includes details about the dissolved protein, such as its concentration and possible PDB-structure code [7]. In addition, information is given on the chemical identity of the buffer, its pH value, the ionic strength of the solution, the temperature, and the possible presence of different excipients (co-solutes) is also taken into account. In its current version, BioMThermDB 1.0 provides several protein-solution properties that are important for determining their stability, such as the hydrodynamic radius, electrophoretic mobility, zeta potential, and the so-called cloud-point temperature, which is the point at which protein solutions separate into two co-existing phases [8,9,10]. A piece of indispensable information, especially for modeling protein solutions, is also the viscosity of the solvent and protein solution itself [11]. In addition, each entry contains the details of the experimental technique used to obtain the thermophysical data of a certain protein solution, as well as the DOI of the corresponding original article in which the results were first published.

**Figure 2:**
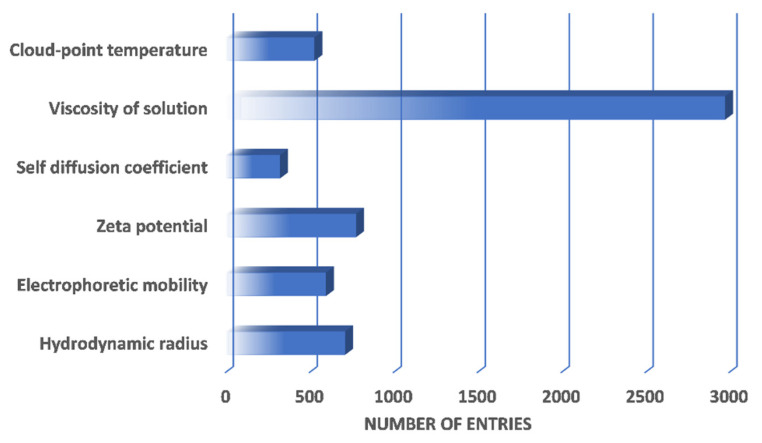
Distribution of thermophysical data entries based on measured properties.

BioMThermDB 1.0 currently is comprised of 5889 specific entries, of which 77.4% belong to globular and other proteins, and the remaining 22.6% are represented by anti-bodies. More than half of all listed protein solutions have their viscosity measured (Figure 2), which makes them very useful in terms of designing protein formulations and verifying calculated results. Figure 2 also reveals that the database already contains at least 500 entries in almost every physical-feature category (the exception being the self-diffusion coefficient) and it will continue to grow further.

**Figure 3:**
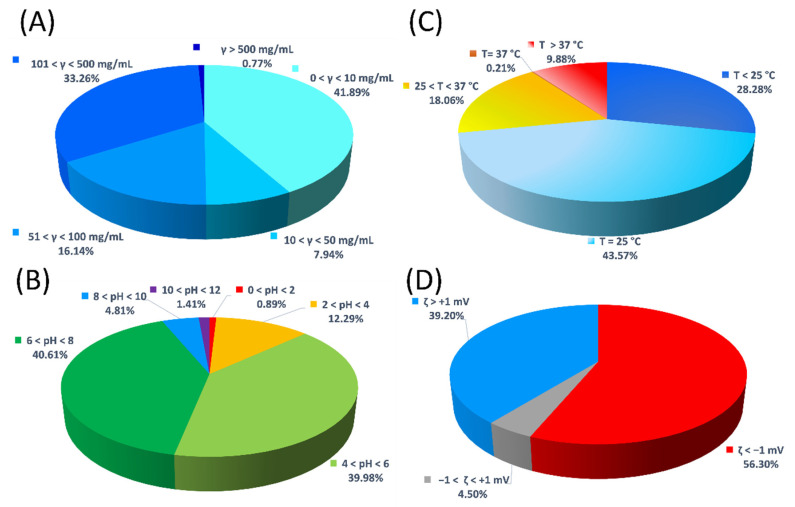
Different distributions of protein thermophysical and thermodynamic data based on (**A**) concentration of protein solutions, (**B**) pH value of protein solutions, (**C**) temperature of protein solutions, and (**D**) zeta potential of protein solutions.

Figure 3A demonstrates that the concentration ranges of our database span over all areas, from almost completely diluted to extremely concentrated protein solutions. However, most entries are found in two ranges, namely between 0 and 10, and 101 and 500 mg mL^−1^, as they together represent 75.15% of all the data. This is due to the well-known fact that interparticle interactions are usually studied in dilute systems; on the other hand, experiments for observing, e.g., liquid–liquid phase separation, are mostly performed at concentrations above 90 mg mL^−1^ [12,13,14].

Similar to concentration regimes, BioMThermDB 1.0 covers protein thermophysical data throughout the whole pH range, with experiments carried out even in the harshest known conditions (pH below 2 and above 10), as displayed in Figure 3B. Of course, the majority of entries are in the vicinity of physiological conditions (i.e., 40.61% of entries are between pH = 6 and 8) since they are most important for studying various properties of protein solutions, with an emphasis on the formulations of biological drugs. In terms of pH and buffers, this database is of additional value considering that, given the pH values in combination with buffer identity and the ionic strength of solution, one can more easily shed light on often underestimated buffer-specific effects that could be incorporated into results. Buffers can govern many aspects of protein stability, e.g., conformational, colloidal, and interfacial stability, and, as such, are a non-negligible part of protein solutions [13,15].

Regarding temperature, one can find most entries (43.57%) at room temperature (Figure 3C) considering that working with those temperatures usually presents the lowest probability of early-protein aggregation onset. However, one is sometimes in search of quite the opposite, namely in the case of when one seeks out the occurrence of protein self-assembly. The self-association of proteins can be achieved by both the cooling and heating of protein solutions. Many such experiments are represented by the database entries whose properties are determined below 25 (28.28%) and above 37 °C (9.88%). The cooling of protein solutions is usually involved in cloud-point measurements, while heating is often necessary for in vitro onset of protein fibrillization [16,17].

Another important physical property that can be used to shorten the time needed to produce trial-protein formulations and help one to both optimize and assess their long-term stability is the zeta potential. The distribution of this indicator of the protein surface charge, as depicted in Figure 3D, shows that only 4.5% of all entries have a zeta potential in the range between −1 and +1 mV, which marks the least stable and most aggregation-prone protein solutions. Protein formulations dominated by repulsive interactions are more stable and, among the entries in BioMThermDB 1.0, these are represented with a pronounced negative (56.30%) or positive (39.20%) zeta potential.

## 3. Materials and Methods

BioMThermDB 1.0 is currently developed using the HTML programming language and is freely accessible at https://phys-biol-modeling.fkkt.uni-lj.si/biomthermdb.html (current version of 5 October 2022). The editing and ordering of BioMThermDB 1.0 entries, their statistical analysis (calculation of percentages and counting of data), and the final visualization were performed in Microsoft Excel. At the moment, BioMThermDB 1.0 is in tabular form but the database will continue to grow and be upgraded. The development of an efficient data browser is also planned for it in the near future; it will be maintained on a regular basis and both all novelties and upgrades will be published on the BioMThermDB 1.0 homepage.

## 4. Conclusions

To understand and model the stability of protein solutions, a wide and diverse set of information on the thermophysical and thermodynamic properties of proteins in aqueous solutions is of critical importance. Despite the fact that the thermophysical properties of protein solutions are widely studied, the data are scattered in literature and often not consistent due to different protein batches and different experimental techniques. The BioMThermDB 1.0 database presents an overview of the existing published data and some of our own unpublished thermodynamic and thermophysical data on protein solutions that should help scientists in the theoretical treatment of these systems, as well as help experimentalists in planning new experiments.

## Figures and Tables

**Table 1:** List of protein entries for group Other in Figure 1.

Protein	Number of Entries
Soy-protein isolate	107
Rice-flour proteins	42
Erythrocytes	30
Horse globulins	26
CP12C75S (C-terminal disulfide bridge mutant)	26
Globulin	24
Conalbumin	19
CP12C31S (N-terminal disulfide bridge mutant)	18
Microtubule-associated Proteins (MAPs)	14
4S *α*2–*β*1–glycoprotein	13
Recombinant p53 (1–93)	13
Neurofilaments	11
Serum orosomucoid	10
Fibrinogen	10
Lipoprotein ([4-14C] cholesterol-labeled) in dog’s blood serum	10
Wild-type CP12 protein	7
Ovomucoid O	7
Nuclease	6
*βL*-crystallin	1

## Data Availability

Data is available within the article and on the database web-page.
